# Differential Targeting of c-Maf, Bach-1, and Elmo-1 by microRNA-143 and microRNA-365 Promotes the Intracellular Growth of *Mycobacterium tuberculosis* in Alternatively IL-4/IL-13 Activated Macrophages

**DOI:** 10.3389/fimmu.2019.00421

**Published:** 2019-03-19

**Authors:** Ousman Tamgue, Lorna Gcanga, Mumin Ozturk, Lauren Whitehead, Shandre Pillay, Raygaana Jacobs, Sugata Roy, Sebastian Schmeier, Malika Davids, Yulia A. Medvedeva, Keertan Dheda, Harukazu Suzuki, Frank Brombacher, Reto Guler

**Affiliations:** ^1^International Centre for Genetic Engineering and Biotechnology, Cape Town Component, Cape Town, South Africa; ^2^Division of Immunology and South African Medical Research Council Immunology of Infectious Diseases, Department of Pathology, Faculty of Health Sciences, Institute of Infectious Diseases and Molecular Medicine, University of Cape Town, Cape Town, South Africa; ^3^Department of Biochemistry, Faculty of Sciences, University of Douala, Douala, Cameroon; ^4^RIKEN Center for Integrative Medical Sciences, Yokohama, Japan; ^5^Institute of Natural and Mathematical Sciences, Massey University, Auckland, New Zealand; ^6^Centre for Lung Infection and Immunity, Department of Medicine and UCT Lung Institute, Division of Pulmonology, University of Cape Town, Cape Town, South Africa; ^7^Research Center of Biotechnology, Institute of Bioengineering, Russian Academy of Science, Moscow, Russia; ^8^Department of Computational Biology, Vavilov Institute of General Genetics, Russian Academy of Science, Moscow, Russia; ^9^Department of Biological and Medical Physics, Moscow Institute of Physics and Technology, Dolgoprudny, Russia; ^10^Faculty of Infectious and Tropical Diseases, Department of Immunology and Infection, London School of Hygiene and Tropical Medicine, London, United Kingdom; ^11^Faculty of Health Sciences, Wellcome Centre for Infectious Diseases Research in Africa, Institute of Infectious Disease and Molecular Medicine, University of Cape Town, Cape Town, South Africa

**Keywords:** microRNA-143, microRNA-365, alternative activated macrophages, *Mycobacterium tuberculosis*, CAGE transcriptomics, c-Maf, Bach-1, Elmo-1

## Abstract

*Mycobacterium tuberculosis* (Mtb) can subvert the host defense by skewing macrophage activation toward a less microbicidal alternative activated state to avoid classical effector killing functions. Investigating the molecular basis of this evasion mechanism could uncover potential candidates for host directed therapy against tuberculosis (TB). A limited number of miRNAs have recently been shown to regulate host-mycobacterial interactions. Here, we performed time course kinetics experiments on bone marrow-derived macrophages (BMDMs) and human monocyte-derived macrophages (MDMs) alternatively activated with IL-4, IL-13, or a combination of IL-4/IL-13, followed by infection with Mtb clinical Beijing strain HN878. MiR-143 and miR-365 were highly induced in Mtb-infected M(IL-4/IL-13) BMDMs and MDMs. Knockdown of miR-143 and miR-365 using antagomiRs decreased the intracellular growth of Mtb HN878, reduced the production of IL-6 and CCL5 and promoted the apoptotic death of Mtb HN878-infected M(IL-4/IL-13) BMDMs. Computational target prediction identified c-Maf, Bach-1 and Elmo-1 as potential targets for both miR-143 and miR-365. Functional validation using luciferase assay, RNA-pulldown assay and Western blotting revealed that c-Maf and Bach-1 are directly targeted by miR-143 while c-Maf, Bach-1, and Elmo-1 are direct targets of miR-365. Knockdown of c-Maf using GapmeRs promoted intracellular Mtb growth when compared to control treated M(IL-4/IL-13) macrophages. Meanwhile, the blocking of Bach-1 had no effect and blocking Elmo-1 resulted in decreased Mtb growth. Combination treatment of M(IL-4/IL-13) macrophages with miR-143 mimics or miR-365 mimics and c-Maf, Bach-1, or Elmo-1 gene-specific GapmeRs restored Mtb growth in miR-143 mimic-treated groups and enhanced Mtb growth in miR-365 mimics-treated groups, thus suggesting the Mtb growth-promoting activities of miR-143 and miR-365 are mediated at least partially through interaction with c-Maf, Bach-1, and Elmo-1. We further show that knockdown of miR-143 and miR-365 in M(IL-4/IL-13) BMDMs decreased the expression of HO-1 and IL-10 which are known targets of Bach-1 and c-Maf, respectively, with Mtb growth-promoting activities in macrophages. Altogether, our work reports a host detrimental role of miR-143 and miR-365 during Mtb infection and highlights for the first time the role and miRNA-mediated regulation of c-Maf, Bach-1, and Elmo-1 in Mtb-infected M(IL-4/IL-13) macrophages.

## Introduction

Our understanding of the molecular mechanisms driving *Mycobacterium tuberculosis* (Mtb) survival and persistence have contributed significantly to the control of Mtb infection, yet tuberculosis (TB) nevertheless killed an estimated 1.7 million people in 2016 globally, surpassing HIV/AIDS to become the highest cause of mortality from a single infectious agent worldwide (1). This alarming situation urges for continuous and accelerated investigation of the molecular basis of Mtb persistence and evasion of the host immune response. It is known that Mtb hijacks several host factors to evade the effector killing functions of classically (LPS/IFNy) activated macrophages (2–5) into the less microbicidal alternatively (IL-4/IL-13) activated macrophages (6). Such host factors include microRNAs (miRNAs) which are an abundant class of highly conserved small (~22 nucleotides) non-coding RNAs that inhibit the expression of their target genes mainly by binding complementary regions in the 3′ untranslated region (3′UTR) of mRNAs (7). Several circulating miRNAs have been detected in active pulmonary tuberculosis patients; however, a clear relationship between pathological and physiological conditions is still missing (8–11). Functional roles of a limited number of deregulated miRNAs during Mtb infection of mice have been reported. For instance, miR-223 depletion rendered TB resistant C57BL/6 mice highly susceptible to infection and subsequent death. In addition, miR-223 targets several chemo-attractants in myeloid cells thereby controlling lung recruitment of these cells and subsequently promotes neutrophil-driven lethal inflammation (12). Inhibition of macrophage apoptosis is another well-known mechanism by which Mtb evades the host defense (13). MiR-582-5p, up-regulated in active TB patients, was found to inhibit apoptosis in monocytes through negative regulation of FOXO1 (14). In contrast, miR-155 promoted *M. bovis* BCG-mediated apoptosis of macrophages (15) whereas miR-155 promoted autophagy in Mtb-infected macrophages (16).

MiR-143 and miR-365 have been associated with immune regulation. MiR-143 for instance targets toll-like receptor 2 expression in colorectal carcinoma cell lines to inhibit tumor invasion and migration (17). In addition, miR-143 targets transforming growth factor-β activating kinase 1 (TAK1) and modulates MAPK and NF-κB signaling pathways in pancreatic ductal adenocarcinoma cells (18). Furthermore, miR-143 was downregulated in M(IFNγ/LPS) when compared to M(IL-4) polarized murine macrophages (19). MiR-365 was reported to be a negative regulator of IL-6 expression in Hela and HEK cell lines (20). Also, miR-365 reportedly targets the serum and glucocorticoid-regulated kinase 1 (SGK1) to regulate MDM2/p53 expression, cell cycle progression and apoptosis in trophoblasts (21). MiR-365 was further reported to promote apoptosis of retinal neurons in diabetic rats by targeting IGF-1 (22).

Here we identified miR-143 and miR-365 which were highly up-regulated in Mtb-infected M(IL-4/IL-13) polarized macrophages. Knockdown of miR-143 and miR-365 decreased mycobacterial burden and significantly reduced the release of chemokine CCL5 and pro-inflammatory cytokine IL-6 in M(IL-4/IL-13) polarized macrophages. We then validated c-Maf, Bach-1 and Elmo-1 as potential target genes for both miRNAs and demonstrated that miR-143 and miR-365 functions are mediated at least partially through interaction with c-Maf, Bach-1, and Elmo-1. Taken together, our data suggest miR-143 and miR-365 upregulation plays a host-detrimental role during Mtb infection of M(IL-4/IL-13) macrophages, and establish for the first time that the activities of miR-143 and miR-365 are mediated by the direct targeting of c-Maf, Bach-1 and Elmo-1 in Mtb-infected M(IL-4/IL-13) macrophages.

## Materials and Methods

### Generation of Bone Marrow-Derived Macrophages (BMDMs)

Bone marrow-derived macrophages were generated from 8 to 11 weeks old male BALB/c mice. The mice were sacrificed using halothane and death was confirmed by cervical dislocation. The mice femur bones were flushed using plain Dulbecco's modified Eagle's medium (DMEM) (Gibco, Invitrogen Corporation, Carlsbad, CA, USA) to collect (centrifugation: 1,200 rpm; 4°C; 10 min) bone marrow cells. The cells were seeded in sterile 100 mm CORNING plates (14 × 10^6^ cells/ml) and incubated (37°C; 5% CO_2_; 70% RH) for 10 days in PLUTZNIK media (DMEM containing 10% Fetal Calf Serum, 5% horse serum, 2 mM L-glutamine, 1 mM Na-pyruvate, 0.1 mM 2-beta-Mercaptoethanol, 30% L929 cell-conditioned medium, 100 U/ml penicillin G, 100 μg/ml streptomycin) to allow for their differentiation into BMDMs. On day 10, the adherent cells were lifted using 4 mg/ml Lidocaine EDTA solution and gentle scraping, thereafter washed twice with DMEM (containing 10% FCS, 100 U/ml penicillin G, 100 μg/ml streptomycin) to remove residual M-CSF (present in the L929 conditioned medium). The BMDMs were seeded at 2–3 × 10^6^ cells/well, 5 × 10^5^ cells/well and 1 × 10^5^ cells/well in 6, 24, and 96-well Nunc plates, respectively and incubated (37°C; 5% CO_2_; 70% RH) overnight before performing downstream experiments.

### Generation of Peripheral Blood Mononuclear Cells (PBMCs) and Monocyte-Derived Macrophages (MDMs)

PBMCs were isolated using Histopaque®-10771 (Sigma-Aldrich Biotechnology LP and Sigma-Aldrich Co., St Louis, MO) density gradient centrifugation. Blood was collected in VACUETTE® K3E blood collection tubes (Greiner Bio-one, GmbH, Germany), transferred to 50 ml Falcon tubes and diluted 1:2 with isotonic phosphate buffered saline (PBS) solution pH 7.4. Thirty ml of diluted blood was transferred to 50 ml Leucosep™ 227290 tubes (Greiner Bio-one, GmbH, Germany) loaded beforehand with 15 ml Histopaque® solution as per the manufacturer instructions. The tubes were centrifuged at 1,000 × g (2,229 rpm) for 25 min (acceleration: 9; deceleration: 0) using an Eppendorf® 5810R centrifuge (A-4-81 Rotor, radius 18 cm). Plasma was discarded and buffy coat (lymphocytes/PBMCs) layer was harvested into a fresh 50 ml Falcon tube. The buffy coat was washed 3 times with 50 ml PBS solution pH 7.4 and spun at 250 × g for 10 min (acceleration 9, deceleration 5). PBMCs were re-suspended in 2–5 ml complete growth medium (RPMI 1640 medium supplemented with 10% FCS, 2 mM L-glutamine and 1% penicillin G/streptomycin. All purchased from Life Technologies™, Carlsbad, CA, USA). PBMCs were plated at 1 × 10^5^ cells per 96-well tissue culture plates (Corning Costar®, Cambridge, MA) in complete growth medium for downstream bacterial burden experiments.

For the generation of MDMs, PBMCs were plated in 6-/12-/96-well tissue culture plates (Corning Costar®, Cambridge, MA) at a density of 20/10/1 × 10^6^ cells per well respectively and incubated (37°C; 5% CO_2_; 70% RH) for 2 h to allow monocytes to adhere. Non-adherent cells were discarded, and adherent monocytes were given a gentle wash with PBS then incubated in X-VIVO™ 15 serum free hematopoietic medium (supplemented with 1% penicillin G/streptomycin) for 7 days to allow for the differentiation of monocytes into MDMs. X-VIVO™ 15 serum free hematopoietic medium was changed on day 4. On day 7, X-VIVO™ 15 was removed, MDMs washed once with PBS and complete growth medium added for downstream experiments. MDMs purity was assessed by fluorescence-activated cell sorting (FACS) analysis using a PE–labeled anti-CD11b; PerCP–labeled anti-HLA-DR; FITC–labeled anti-CD14 and APC–labeled anti-CD3 monoclonal antibodies (all purchased from BD Biosciences™ CA, USA). MDMs purity exceeded 95%.

### Culture of RAW264.7 Murine Macrophage Cell Line

The murine macrophage cell line RAW264.7 were purchased from ATTC. The cells were cultured in an incubator (37°C; 5% CO_2_; 70% RH) using DMEM medium supplemented with 10% Fetal Calf Serum, 4 mM glutamine and penicillin/streptomycin (all purchased from Life Technologies™, Carlsbad, CA, USA).

### Transfection of Power Inhibitors (AntagomiRs) microRNAs Mimics and Genes LNA Antisense-Oligonucleotides (GapmeRs)

MiR-143-3p and miR-365a-3p MiRCURY LNA™ microRNA Power Inhibitor*s* (AntagomiRs), mimics and GapmeRs (Exiqon™/Qiagen, Germany) were diluted in Opti-MEM medium (Life Technologies™, Carlsbad, CA, USA) and mixed in a 1:1 ratio with Lipofectamine 3000 (Life Technologies™, Carlsbad, CA, USA). Cells were transfected with 25 nM of the antagomiR/mimic/GapmeR:lipofectamine solution per well for 24 or 48 h, depending on the duration and plate format required for downstream experiments. In the case of double transfection, the miR-mimics and the GapmeRs were transfected in a 1:1 ratio at a final concentration of 25 nM each for the miR-mimics and the GapmeRs.

### Stimulation and *Mycobacterium tuberculosis* Infection of BMDMs and MDMs

Macrophages were stimulated with recombinant mouse (100 units/ml) or recombinant human (10 ng/ml) IL-4, IL-13 or a combination of IL-4 and IL-13 (IL-4/13) (BD Bio-sciences). At 24 h post-stimulation, macrophages were infected with Mtb strain HN878 (clinical hypervirulent strain) at a multiplicity of infection (MOI) of 5 bacilli: 1 cell (5:1) in fresh DMEM containing 10% FCS with no antibiotics (penicillin G/streptomycin). After 4 h, the supernatant was removed and fresh DMEM containing 10% FCS, 100 U/ml penicillin G, 100 μg/ml streptomycin, 10 ug/ml Gentamicin with or without stimulants was added to remove extracellular bacteria. Two hours later, the medium was replaced with DMEM containing 10% FCS, 100 U/ml penicillin G, 100 μg/ml streptomycin, with or without stimulants. Included in an individual experiment, BMDMs were also infected with the laboratory strain Mtb H37Rv for comparison of host response to the clinical hypervirulent Mtb strain HN878.

### RNA Extraction

Cells were lysed in Qiazol (Qiagen, Germany) at different time points post-transfection/-stimulation/-infection and lysates were stored at −80°C. Total RNA was isolated from the lysate using miRNeasy Mini kit (Qiagen, Germany) according to the manufacturer's instructions. RNA quantity and purity were measured using the ND-1000 NanoDrop spectrophotometer (ThermoScientific, DE, USA).

### cDNA Synthesis and RT-qPCR

For protein coding gene expression analysis, 100 ng total RNA was reverse-transcribed into cDNA using Transcriptor First Strand cDNA Synthesis Kit (Roche, Germany) according to the manufacturer's instructions. Quantitative real-time PCR (RT-qPCR) was performed using LightCycler® 480 SYBR Green I Master (Roche, Germany) and gene-specific primers (IDT, CA, USA). Fold change in gene expression was calculated by the ΔΔCt method and normalized to Hprt1 which was used as internal control. The non-stimulated 0 h sample was set to 1. With regard to miRNAs, total RNA was diluted to 5 ng/μl before cDNA was synthesized using miRCURY™ LNA MicroRNA PCR System First-Strand cDNA Synthesis Kit (Exiqon, Denmark) as per the manufacturer's instructions. RT-qPCR was performed using miRCURY LNA™ Universal RT microRNA PCR New ExiLENT SYBR® Green Master Mix and microRNA *LNA*™ PCR primer sets for miR-365a-3p, miR-143-3p and miR-191 as internal control (Exiqon, Denmark) in a Roche 480 Lightcycler (Roche, Germany). Fold expression was calculated using the ΔΔCt method with non-stimulated 0 h sample set as 1 (calibrator). Protein coding gene and microRNA qPCR primer sequences and PCR run conditions are listed in Supplementary Table S1.

Several internal controls including U6, 5S, miR-423-3p, and miR-191-3p were tested before hand in order to select a suitable reference for normalization of target miRNA expression. U6, 5S, miR-423-3p, and miR-191-3p expression levels were analyzed during IL-4, IL-13, and IL-4/13 stimulation with or without Mtb infection included in the experiment procedure. Expression levels were compared to each other and analyzed using the bestkeeper algorithm. MiR-191-3p was the most stably expressed during IL-4, IL-13, and IL-4/IL-13 stimulation with Mtb infection included, based on its low standard deviation of crossing point (Cp) value. As a result, miR-191-3p was selected as the reference RNA for normalization of target miRNA expression.

### Bacterial Burden Determination

At 24 and 48 h post-infection, supernatant was removed from Mtb-infected macrophages and stored at −80°C for analysis of cytokine production. Cells were lysed at 4 and 24 h post-infection in Triton X-100 and serial dilutions were plated on Middlebrook 7H10 agar plates and incubated for 15 days at 37°C in 5% CO_2_. Colony forming Units (CFUs) were enumerated to determine bacterial burden.

### Cytokine Production

Cytokine production was measured by enzyme-linked immunosorbent assay (ELISA) using ELISA development reagents (R&D systems, USA). Culture supernatants were diluted 2, 3, and 6-folds. Data was acquired using Versamax™ Tunable microplate reader with Softmax Pro v6.3 (Avantor®, US).

### Prediction of miR-143 and miR-365 Target Genes

To identify the direct targets genes of miR-143 and miR-365, we used free online miRNA-target predictions algorithms (miRanda, Target Scan, PicTar, using default parameters) as well as IRNdb, a webserver we developed together with our collaborators to collect known and predicted miRNA target genes (23). From the list of predicted immune relevant target genes, we selected those that were downregulated in IL-4/IL-13 and IL-4/IL-13 + Mtb as compared to non-stimulated conditions in our BMDM CAGE (Cap Analysis of Gene Expression) transcriptomics data using a program developed by our collaborator (http://compbio.massey.ac.nz/apps/tbvis2). We further filtered the target genes showing medium (TPM 20–100) to high (TPM > 100) basal expression level (24). The filtering was based on the assumption that target genes that are highly expressed in macrophages are more likely to play an important functional role in that cell type. Last, we shortlisted target genes predicted by at least 2 two algorithms for miR-143 or miR-365.

### Cloning of 3′-UTR, Site-Directed Mutagenesis and Luciferase Assay

Validation of miR-143 and miR-365 targets c-Maf (ENSMUST00000109104), Bach-1 (ENSMUST00000026703) and Elmo-1 (ENSMUST00000072519) was performed by cloning an ~500 bp portion of the 3′-UTRs encompassing the miRNA binding sites. PCR products were amplified using primers with restriction sites for XbaI and SacI and cloned into the pmiRGLO vector (Promega, US). The 3′-UTR-vector constructs were sequenced to verify the correct alignment of the 3′-UTR sequence and the absence of mutations. For luciferase reporter assays, Raw264.7 cells were seeded into 96-well plates at a density of 4 × 10^4^ cells/well, then co-transfected with 100 ng of 3′-UTR-vector/empty vector, together with 10 nM scramble (NC) or miR-143/miR-365 mimics for 72 h. Luciferase activity was measured using the Dual-Glo®Luciferase Assay System (Promega, US) according to the manufacturer's instructions. Firefly luciferase activity (F-luc) was normalized to Renilla luciferase (R-luc) in order to correct for differences in transfection efficiency. Luciferase activity of the 3′-UTR + miR NC group was set to 1. Mutant 3′-UTR luciferase vectors were generated by designing a primer pair harboring a centered 4 point mutation (A to C and T to G) into the complementary seed sequence of miR-143 or miR-365 on the wild-type 3′UTR. Mutagenesis was performed using the Geneart Site Directed Mutagenesis kit A13282 (Thermofisher) as per the manufacturer instructions. The success of the mutagenesis was verified by sequencing.

### RNA Pulldown With Biotinylated Micro-RNAs Mimics

8 × 10^6^ RAW264.7 cells were grown on 10 cm petri dishes until confluency, then transfected with 2 nM final concentration of biotinylated-miR-143/miR-365 mimics. Twenty-four hours later, cells were lysed in 1 ml RIPA buffer and 500 μl lysate were used for precipitation with streptavidin magnetic beads. The precipitates were then used for RNA extraction and qPCR analysis of genes enrichment. A 100 ul (20%) of the cell lysate was saved and used as input.

### Western Blot Analysis

Cells were harvested and lysed in RIPA buffer supplemented with protease inhibitors (P-8340, Sigma-Aldrich, US). Protein concentrations in cell lysates were measured using the Pierce™ BCA protein assay kit (23225, Thermo Fischer Scientific, US), and the same amount of protein was loaded and separated on a polyacrylamide gel. Proteins were transferred onto a nitrocellulose membrane which was subsequently blocked for 2 h with a 2% BSA solution (prepared in TBST) and incubated overnight with primary antibodies against mouse c-Maf (sc-7866), Bach-1(sc-100995), Elmo-1 (sc-166661) and the loading control Gapdh (sc-365062). Membranes were then incubated with horse radish peroxidase (HRP)-conjugated secondary antibodies directed against mouse (sc-2005) or goat (sc-2020) species (all antibodies purchased from Santa Cruz® Technology, CA, US). Membranes were incubated with LumiGlo Reserve™ chemiluminescent substrate kit (54-61-01, Sera care, Life Sciences, MA, US) and protein bands were visualized using the UVP Biospectrum™ 500 imaging system with the VisionWorks® images acquisition and analysis software (Analytik Jena, CA, US).

### Flow Cytometry and Apoptosis

Assessment of apoptosis was measured by flow cytometry using the active caspase-3 as previously reported (25, 26). To discriminate viable from non-viable, cells were stained and incubated in FVS V500 dye (BD Bioscience, US) for 15 min at room temperature. For surface staining, 1 × 10^6^ cells were labeled with appropriate antibodies: MHCII (M5/114.15.2), CD11b (M1/70) and F4/80 (PM8) purchased from BD Biosciences (Fanklin Lakes, NJ, US) and eBioscience (San Diego, CA, US). For intracellular staining, cells were washed, fixed and permeabilized with 1X Fixation-Permeabilization buffer and then stained as per manufacturer's instruction with appropriate antibodies: caspase-3 (C92-605) and Bcl-2 (RUO) (all from BD Biosciences, US). After the incubation period cells were resuspended in FACS buffer for acquisition. Acquisition was performed using BD LSRFortessa (BD Biosciences), and data were analyzed using FlowJo software (Treestar, Ashland, OR, US).

### Transcription Factors Binding Site Analysis

The analysis of transcription factors (c-Maf and Bach-1) binding sites on the promoters of our target genes was done using the online free tool LASAGNA (http://biogrid-lasagna.engr.uconn.edu/lasagna_search/) using the following parameters: Search restricted to mus musculus; cut off *p*-value set to 0.005; score cut off set at 5; reporting of the top 5 TFBS.

### Graphs and Statistical Analysis

Results were plotted using GraphPad Prism v5 and analyzed using an unpaired, two-tailed *t*-test or two-way ANOVA as relevant, with *p*-values represented as *P* > 0.05 ns, ^*^p < 0.05, ^**^p < 0.01, and ^***^p < 0.001.

## Results

### MiR-143 and miR-365 Are Upregulated in Mtb HN878-Infected M(IL-4/IL-13) Activated Murine Macrophages

**Figure 1:**
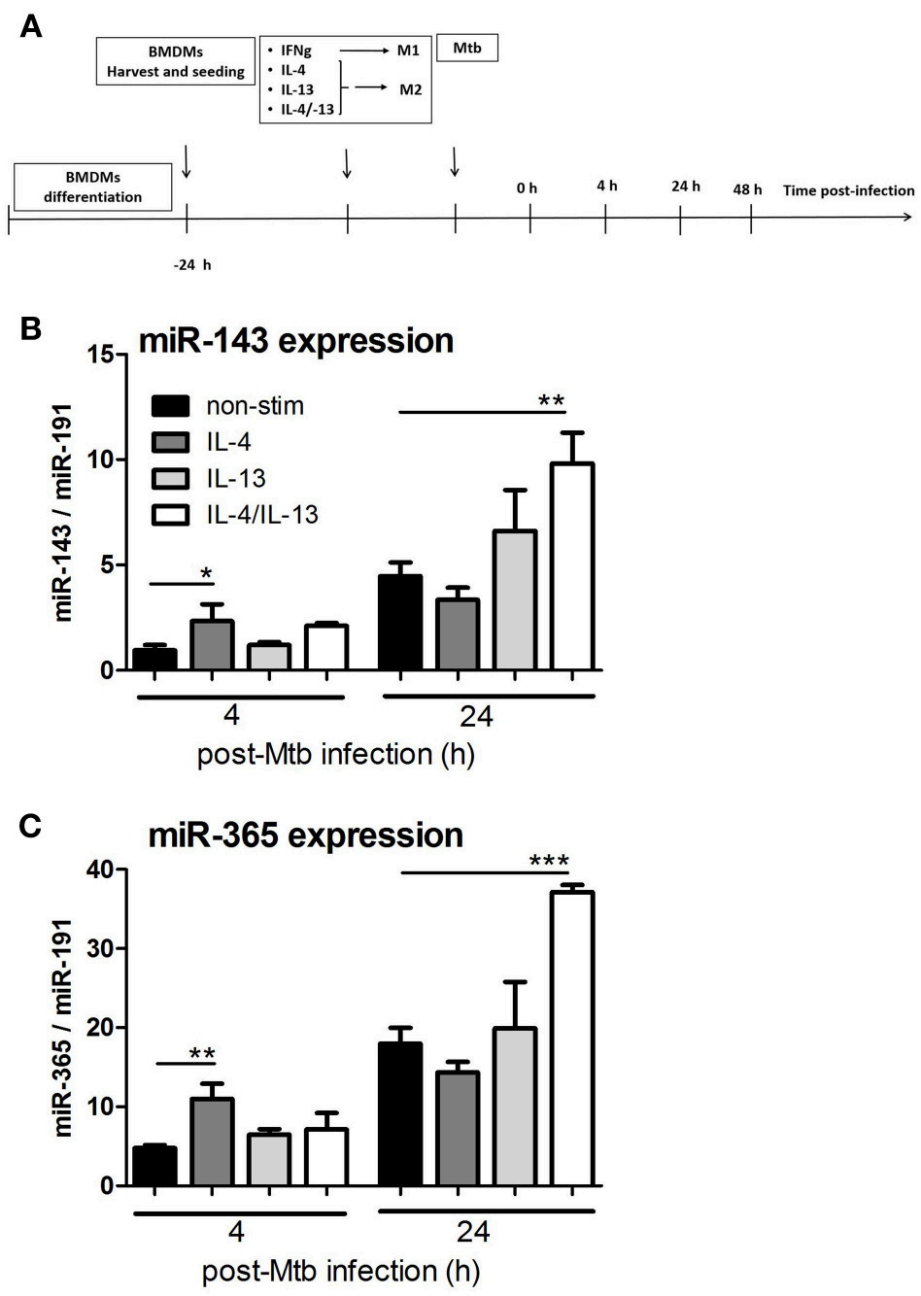
MiR-143 and miR-365 are upregulated in Mtb HN878-infected M(IL-4/IL-13) activated mouse macrophages. **(A)** Timeline of mouse macrophage activation and Mtb HN878 infection. Bone marrow cells were differentiated for 10 days into BMDMs and stimulated with IFNγ or IL-4, IL-13, IL-4/IL-13. At 24 h post-stimulation, BMDMs were infected with Mtb (HN878) for 4, 24, and 48 h. RNA was extracted from lysed cells at different time points post-stimulation and post-Mtb infection. **(B,C)** RT-qPCR analysis of miR-143 and miR-365 kinetic expression in non-infected and Mtb-infected BMDMs. The fold change in gene expression was determined by RT-qPCR and was normalized to miR-191 expression. Non-stimulated 0 h was set to 1. Each data point represents arithmetic mean of triplicates ± SEM. Two-way ANOVA was used to evaluate statistical significance, *P*-values represented as, ^*^P < 0.05, ^**^P < 0.01, and ^***^P < 0.001.

Using deep CAGE transcriptomics, we previously identified several non-coding factors that regulate the polarization of macrophages toward classical or alternative phenotype (27) and during Mtb infection (28). In patients with active pulmonary tuberculosis, mir-143 and miR-365 were highly upregulated in serum when compared to matched healthy controls (8, 29). Expanding on that work, we were interested in understanding the transcriptional regulation of miR-143 and miR-365 during macrophage polarization and Mtb infection. Hence, we performed a time course kinetic experiment using BMDMs stimulated with IL-4, IL-13, and a combination of IL-4 and IL-13 (IL-4/IL-13) for alternative activation, followed by Mtb HN878 infection. RNA samples were collected to measure miR-143 and miR-365 expression by RT-qPCR (Figure 1A). Mtb infection led to a significant increase of miR-143 (Figure 1B) and miR-365 (Figure 1C) expression in M(IL-4/IL-13) stimulated BMDMs at 24 h p.i. when compared to non-stimulated controls. Since the most significant up-regulation of both miR-143 and miR-365 was observed in Mtb-infected M(IL-4/IL-13) BMDMs, therefore IL-4/IL-13 combination was used as alternative macrophage polarizer in our subsequent experiments. Using RT-qPCR we observed that Mtb infection led to increased expression of miR-143 and miR-365 in IL-4/IL-13 stimulated human monocyte derived macrophages (MDMs) at 4 h p.i (Figure S1A). We further observed that miR-143 and miR-365 expression was Mtb strain dependent, with the hypervirulent strain HN878 showing increased miR-143/miR-365 expression as compared to the Mtb lab strain H37Rv (Figure S3C). Taken together, these results show that miR-143 and miR-365 are up-regulated in Mtb-infected M(IL-4/IL-13) alternatively activated macrophages.

### MiR-143 and miR-365 Promote Intracellular Mtb Growth in M(IL-4/IL-13) Polarized Macrophages

**Figure 2:**
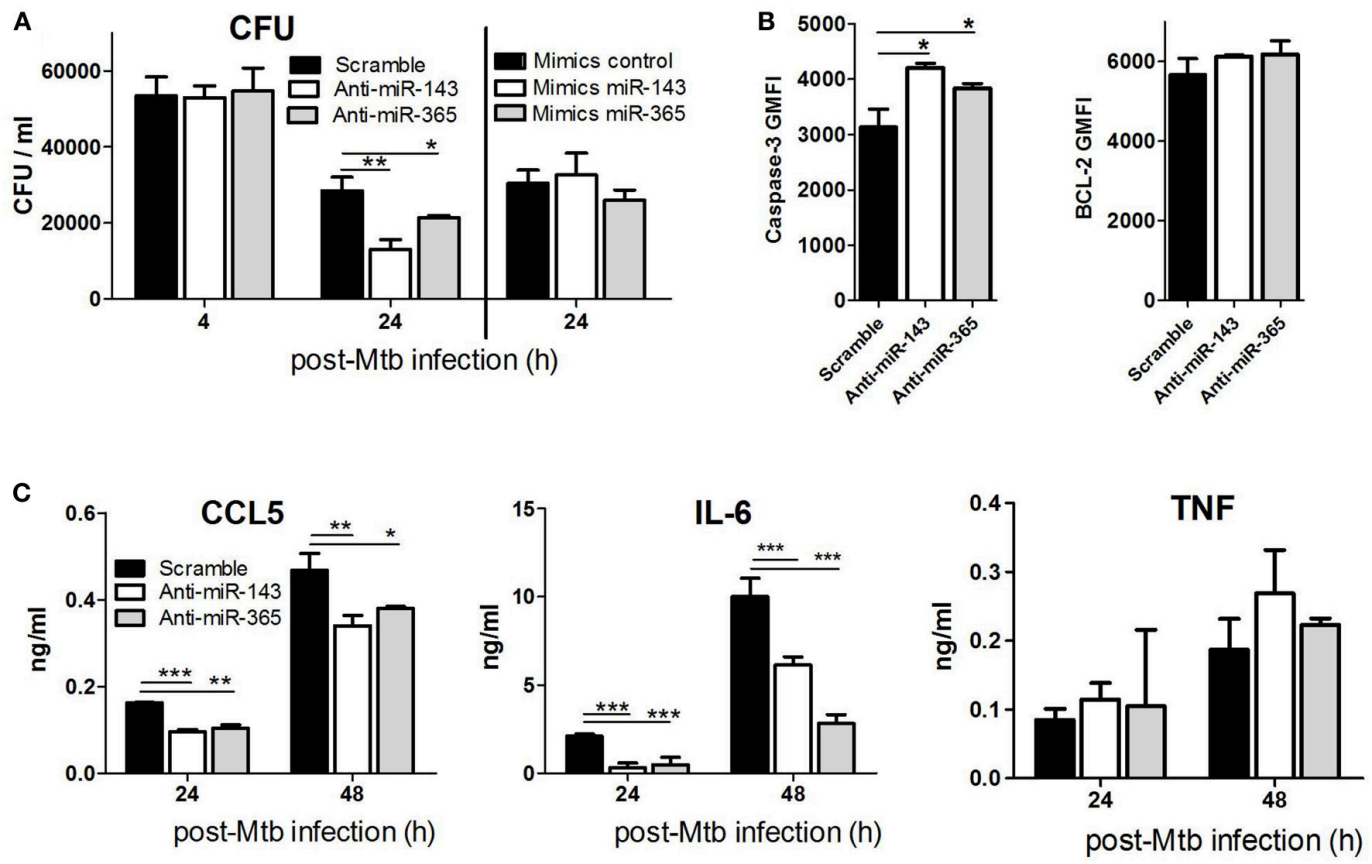
Knockdown of miR-143 and miR-365 decreases intracellular Mtb HN878 growth, CCL5 and IL-6 production and increases apoptotic death of M(IL-4/IL-13) stimulated mouse macrophages. **(A)** BMDMs were transfected with antagomiRs or mimics for miR-143 and miR-365, respectively. Twenty-four hours later, cells were stimulated with IL-4/IL-13 for another 24 h and subsequently infected with Mtb HN878. Cells were lysed at 4 h for uptake and 24 h post-Mtb infection to measure bacterial growth by CFU counting. **(B)** BMDMs were treated as described in **(A)**, cells were harvested and stained with antibodies for active caspase-3 and Bcl2, and analyzed using FACS. **(C)** BMDMs were treated as described in **(A)**. Culture supernatants were analyzed for production of CCL5 chemokine, IL-6 and TNF cytokines at 24 and 48 h post-infection using ELISA. Data represented here are mean ± SD of triplicates. A two-way ANOVA and Bonferroni *post-hoc* test was used to evaluate statistical significance. *P*-values represented as, ^*^P < 0.05, ^**^P < 0.01, and ^***^P < 0.001.

To examine the functional importance of miR-143 and miR-365 in regulating intracellular growth of Mtb in macrophages, we performed a miRNA loss-of-function experiment using chemically synthesized oligonucleotides (antagomiRs). The knockdown of miR-143 and miR-365 by antagomiRs significantly decreased Mtb HN878 growth in M(IL-4/IL-13) stimulated macrophages at 24 h p.i. (Figure 2A). The phagocytic uptake of Mtb at 4 h post-infection was not affected by the knockdown of either miR-143 or miR-365 in M(IL-4/IL-13) stimulated BMDMs, excluding possible antagomiR-induced cytotoxicity or inconstant uptake due to antagomiR treatment. Concordant with the murine macrophage data, M(IL-4/IL-13) polarized human MDMs also exhibited a significantly reduced intracellular growth of Mtb HN878 upon miR-143 and miR-365 knockdown at 24 h post-infection (Figure S1B). Similar to the mouse data, the phagocytic uptake of Mtb was not changed by the miR-143 or miR-365 knockdown in M(IL-4/IL-13) MDMs. We also performed a miRNA gain-of-function experiment using Locked Nucleic Acid (LNA) Antisense oligonucleotides (referred to as GapmeRs). Contrary to the knockdown experiment, overexpression of miR-143 and miR-365 alter Mtb HN878 growth in M(IL-4/IL-13) macrophages at 24 h p.i. (Figure 2A). Together, these data revealed that both miR-143 and miR-365 promote Mtb growth in M(IL-4/IL-13) polarized murine and human macrophages.

### MiR-143 and miR-365 Enhance the Survival of M(IL-4/IL-13) Macrophages by Interfering With Mtb-Infected Cells Apoptosis

To examine whether miR-143 and miR-365-mediated promotion of Mtb intracellular growth is associated with the control of bactericidal cell death pathways, we knocked down miR-143 and miR-365 in M(IL-4/IL-13) BMDMs prior to Mtb infection and measured apoptotic cells by caspase-3 expression using flow cytometry. The knockdown of miR-143 and miR-365 led to a significant increase of apoptotic M(IL-4/IL-13) BMDMs following Mtb HN878, as suggested by significant increase in caspase-3 expression and no change in the expression of the anti-apoptotic marker Bcl-2 (Figure 2B).

### MiR-143 and miR-365 Induce IL-6 and CCL5 Production in M(IL-4/IL-13) Polarized Macrophages

To determine the functional role of miR-143 and miR-365 in regulating chemokine/cytokine production, BMDMs were treated with antagomiRs, infected with Mtb HN878 and TNFα, IL-6, CCL5 production was measured by ELISA. We focused on IL-6 which is a validated direct target of miR-365 (20) as well as CCL5/RANTES and TNFα which are validated indirect targets of miR-143 (30, 31). Knock-down of miR-143 and miR-365 significantly decreased CCL5 and IL-6 production by Mtb-infected M(IL-4/IL-13) macrophages (Figure 2C). Anti-miR-143 and anti-miR-365 treatment had no effect on TNFα production by Mtb-infected M(IL-4/IL-13) macrophages (Figure 2C). These results suggest that miR-143 and miR-365 promote the production of pro-inflammatory IL-6 and CCL5 in M(IL-4/IL-13) polarized macrophage during Mtb infection.

### c-Maf, Bach-1, and Elmo-1 Are Direct Targets of miR-143 and miR-365 in Macrophages

**Figure 3:**
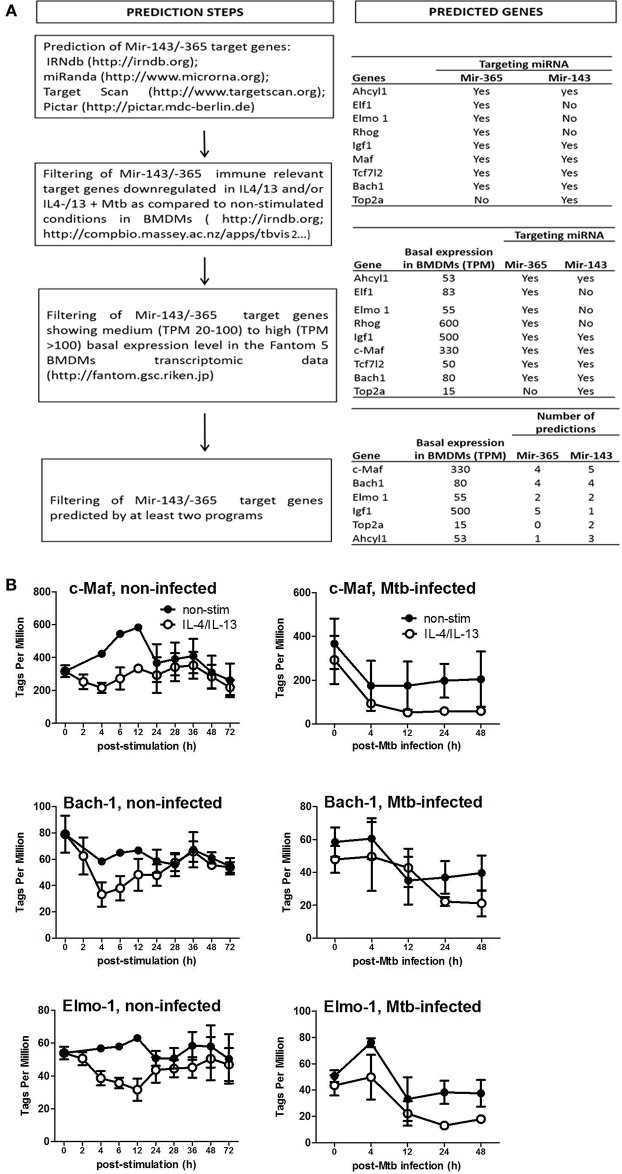
Prediction of miR-143/miR-365 target genes in macrophages. **(A)** Workflow for miR-143/miR-365 target genes prediction. **(B)** MiR-143/miR-365 predicted target genes expression in BMDMs. Data was previously generated in collaboration with the Fantom5 consortium (24) where BMDMs were classically (IFNγ) or alternatively (IL-4 and IL-13) activated then infected with the hypervirulent Mtb HN878 strain for different duration. RNA samples were collected and analyzed by CAGE transcriptomics. Gene expression is quantified as Tags Per Million (TPM).

To identify the direct target genes of miR-143 and miR-365, we used the workflow shown in Figure 3A which combines computational prediction and mining of BMDMs CAGE transcriptomics data, which we previously generated (24). This approach shortlisted c-Maf, Bach-1, Elmo-1, Igf-1, Top2a, and Ahcyl1 as potential target genes of miR-143 and miR-365. We further selected c-Maf, Bach-1 and Elmo-1 for functional validation experiments hence these genes were predicted at least by two miRNA-target predictions algorithms as possible targets for both miR-143 and miR-365 (Figure 3A). Subsequent analysis of CAGE transcriptomics data revealed that c-Maf, Bach-1, and Elmo-1 were downregulated in M(IL-4/IL-13) polarized macrophages when compared to non-stimulated controls, both during stimulation and Mtb infection (Figure 3B).

**Figure 4:**
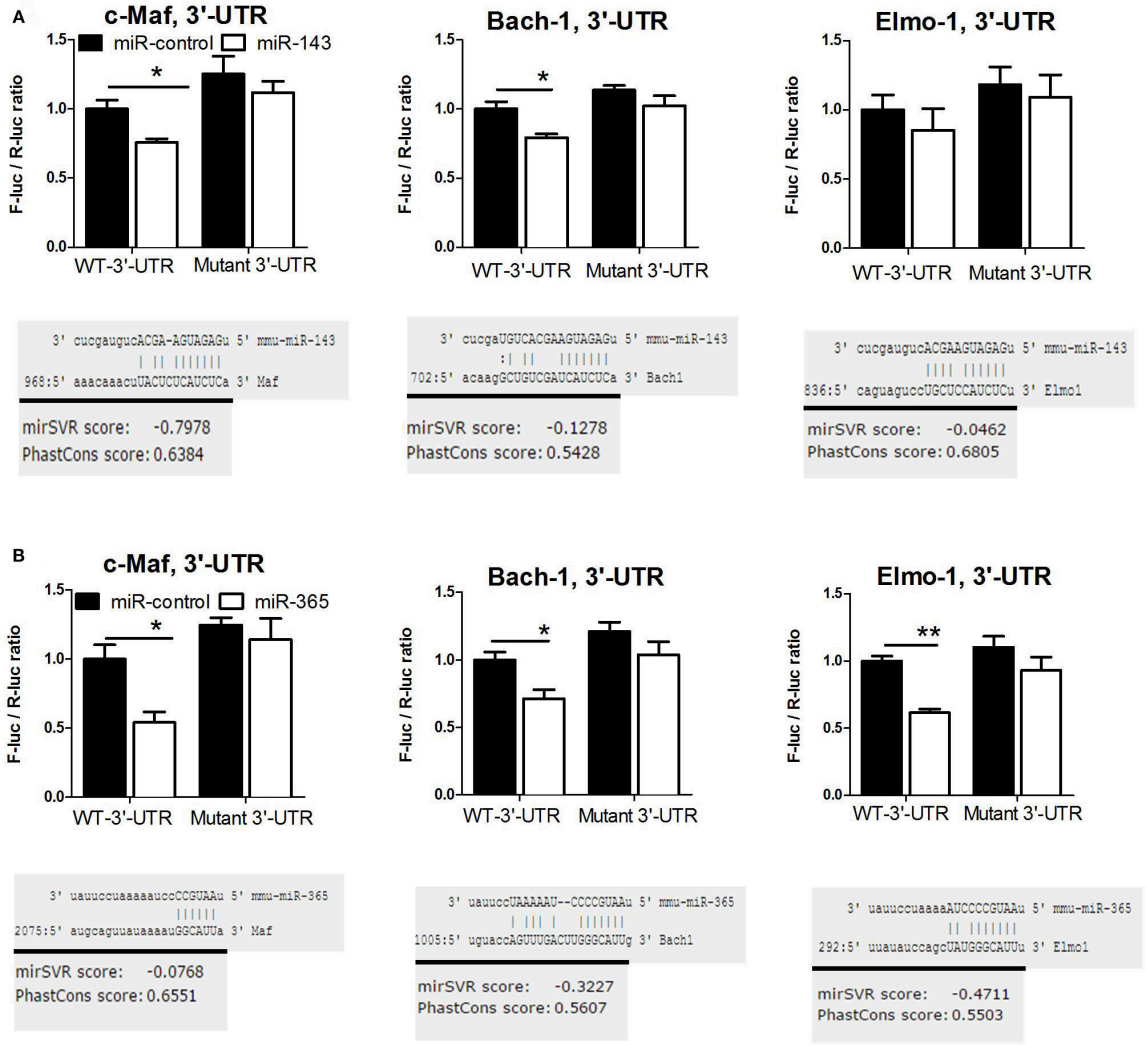
MiR-143 targets c-Maf and Bach-1 while miR-365 targets c-Maf, Bach1, and Elmo-1 in mouse macrophages validated by mutagenesis luciferase assay. Luciferase activity of Raw264.7 cells transfected with reporter plasmid containing the 3′-UTR of c-Maf, Bach1, or Elmo-1 together with **(A)** miR-143 mimic, **(B)** miR-365 mimic or scramble control. At 72 h post-transfection, dual luciferase assay was performed to measure the ratio F-luc/R-luc. Luciferase activity of the 3′-UTR + miR NC group was set to 1. Data represent the mean ± SD of triplicate. Unpaired *t*-test was used to evaluate statistical significance, *P*-values represented as, ^*^P < 0.05 and ^**^P < 0.01.

**Figure 5:**
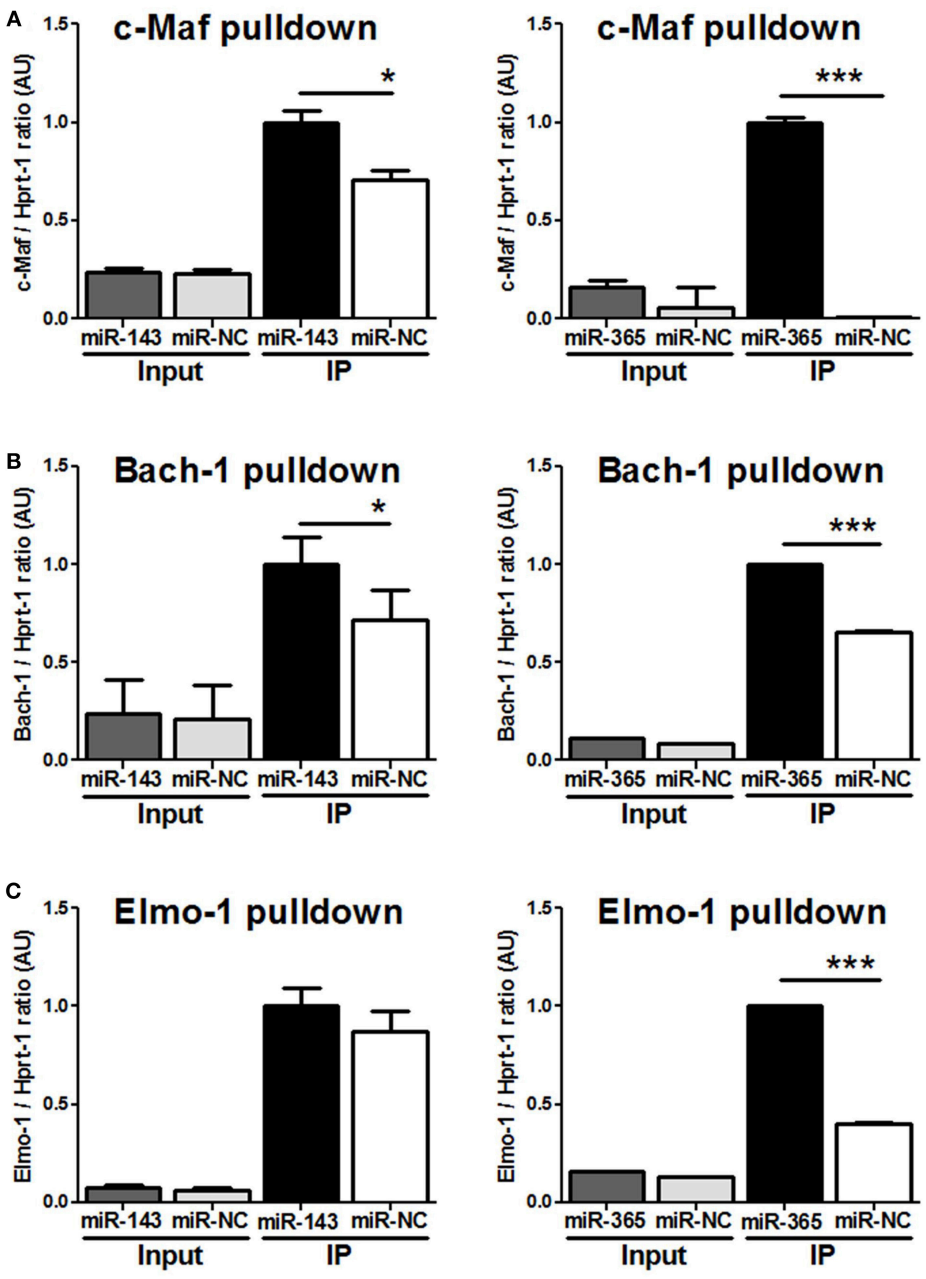
MiR-143 targets c-Maf and Bach-1 while miR-365 targets c-Maf, Bach1, and Elmo-1 in macrophages validated by RNA-pulldown assay. RAW264.7 cells were transfected with biotinylated probes for **(A)** miR-143 mimics or **(B)** miR-365 mimics and whole-cell lysates were precipitated with streptavidin magnetic beads followed by RNA extraction and RT-PCR for c-Maf, Bach-1, and Elmo-1 target genes. 100 ul (20%) of the cell lysate was saved and used as input. Data are the mean ± SD of triplicate. Unpaired *t*-test was used to evaluate statistical significance, *P*-values represented as, ^*^P < 0.05 and ^***^P < 0.001.

To demonstrate that miR-143 and miR-365 directly target c-Maf, Bach-1, and Elmo-1 mRNA, we co-transfected RAW264.7 murine macrophage cells with the reporter plasmid containing the wild-type or the mutant 3′-UTR fragment of the target gene along with either miR-143 mimic or miR-365 mimic to assess luciferase activity (Figure 4). We found a significant decrease of luciferase activity in c-Maf and Bach-1 wild-type 3′-UTR reporter groups in presence of miR-143 (Figure 4A) or miR-365 mimics (Figure 4B), as compared to mimics control-treated groups. MiR-365 mimics significantly decreased luciferase activity in Elmo-1 3′-UTR reporter groups (Figure 4B), while miR-143 mimics had no effect on the luciferase activity of Elmo-1 (Figure 4A). No difference in luciferase activity was observed with all the mimic-treated mutant 3′-UTR. These results suggest that c-Maf, Bach-1 and Elmo-1 are directly targeted by miR-365 while only c-Maf and Bach-1 are direct targets of miR-143. To further confirm the direct targeting of the above genes by miR-365 and miR-143, we transfected RAW264.7 murine macrophage cells with biotin-labeled miR-143 and miR-365 mimics (Figure 5). We then performed RNA pulldown using streptavidin-coated beads and analyzed the pulled-down RNA for the presence of c-Maf, Bach-1, and Elmo-1 transcripts by RT-qPCR. Our results show that miR-143 mimics significantly pulled down c-Maf (Figure 5A) and Bach-1 (Figure 5B) but not Elmo-1 (Figure 5C). MiR-365 mimics significantly pulled down c-Maf, Bach-1 and Elmo-1 (Figures 5A–C). These results further confirm that c-Maf, Bach-1, and Elmo-1 are directly targeted by miR-365 while c-Maf and Bach-1 but not Elmo-1 are directly targeted by miR-143 in murine macrophages.

**Figure 6:**
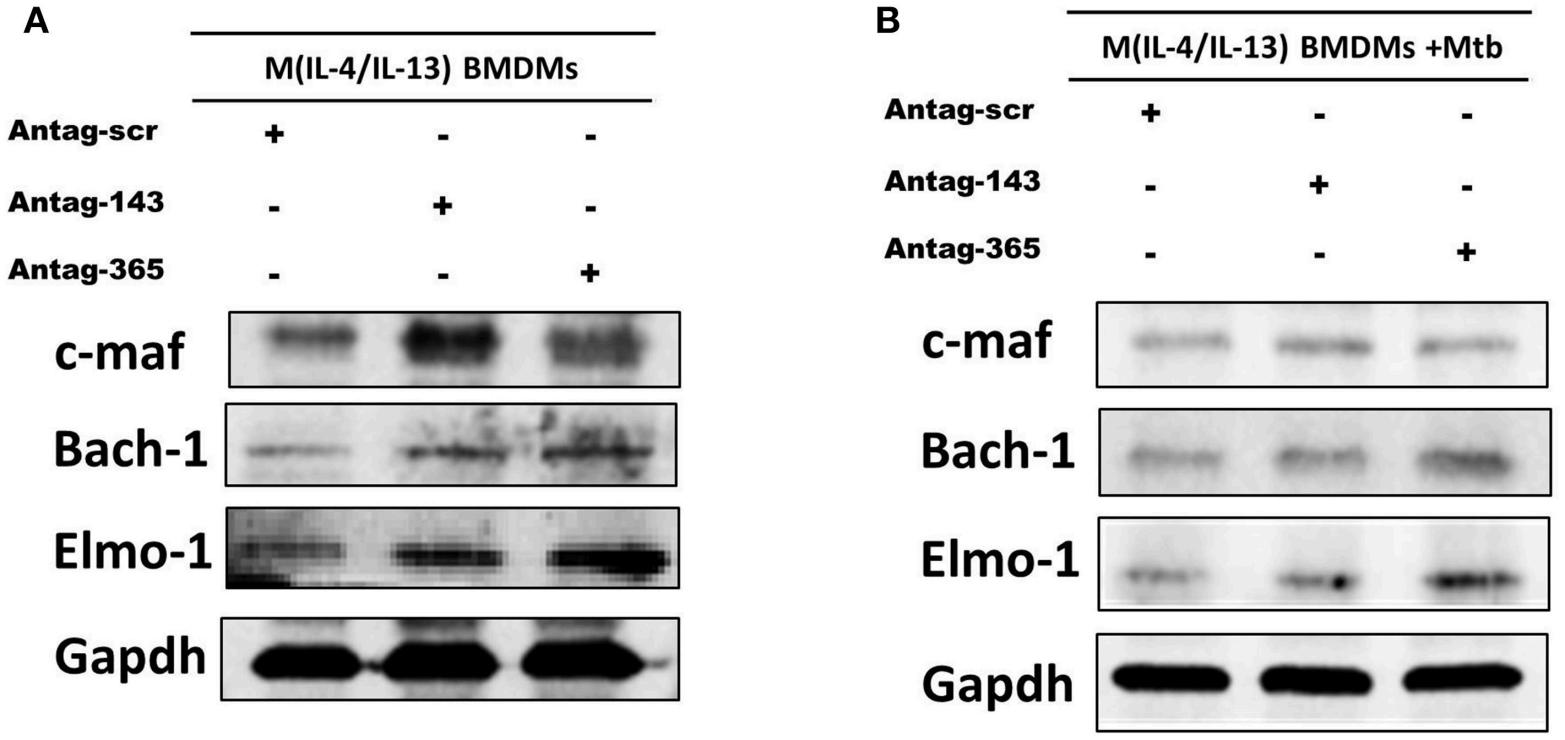
MiR-143 and miR-365 control the protein level of c-Maf, Bach-1, and Elmo-1 in M(IL-4/IL-13) mouse macrophages. **(A)** BMDMs were transfected with antagomiRs scramble, anti-MiR-143, or anti-MiR-365. At 24 h post-transfection, cells were stimulated with IL-4/IL-13 for another 48 h, then total protein was prepared from lysed cells and analyzed by western blotting, using Gapdh protein as internal control for loaded protein amount. **(B)** BMDMs were transfected with antagomiRs scramble, anti-miR-143 or anti-miR-365 for 24 h. Cells were then stimulated with a combination of IL-4/IL-13 for another 24 h and subsequently infected with Mtb HN878 for an additional 24 h. Total protein was prepared from lysed cells and analyzed by western blotting, using Gapdh protein as internal control.

To validate c-Maf, Bach-1 and Elmo-1 as direct targets of miR-143 and miR-365 in a more physiologically relevant condition, we transiently knocked-down these miRNAs in M(IL-4/IL-13) BMDMs using antagomiRs and measured c-Maf, Bach-1, and Elmo-1 protein levels by Western blotting. Knockdown of miR-143 markedly increased protein levels of c-Maf and Bach-1 and to a little extent that of Elmo-1 in M(IL-4/IL-13) BMDMs (Figure 6A). Knockdown of miR-365 resulted in increased c-Maf, Bach-1, and Elmo-1 protein levels in M(IL-4/IL-13) BMDMs (Figure 6A). These results further suggest the direct regulation of c-Maf, Bach-1, and Elmo-1 by miR-143 and miR-365. Interestingly, Mtb infection abrogates the effect of miR-143 and miR-365 antagomiRs treatment on c-Maf, Bach-1, and Elmo-1 protein levels in M(IL-4/IL-13) BMDMs (Figure 6B). These results suggest that Mtb induces a high expression level of miR-143 and miR-365, which overwhelms the knockdown capacity of miR-143 and miR-365 antagomiRs in M(IL-4/IL-13) BMDMs. It might also be possible that Mtb regulates the expression of c-Maf, Bach-1, and Elmo-1 expression through miR-143 and miR-365 unrelated mechanisms. Together, these findings suggest miR-143 and miR-365 differentially control the expression of c-Maf, Bach-1 and Elmo-1 in M(IL-4/IL-13) polarized and Mtb-infected M(IL-4/IL-13) BMDMs.

### c-Maf, Bach-1, and Elmo-1 Regulate Intracellular Mtb Growth and Cytokine/Chemokine Production in M(IL-4/IL-13) BMDMs Through Differential miR-143/miR-365 Regulation

**Figure 7:**
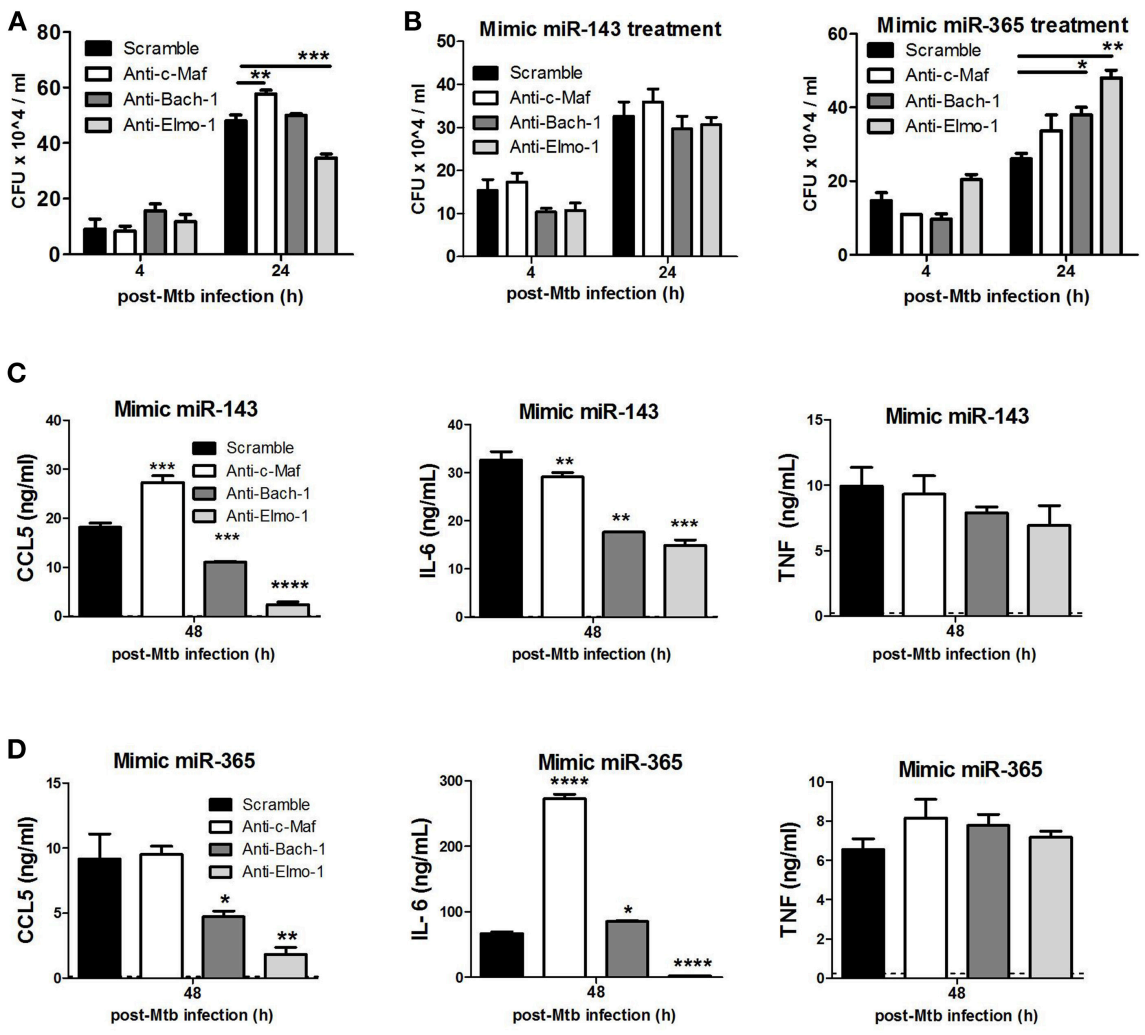
c-Maf, Bach-1, and Elmo-1 regulate Mtb growth, CCL5 and IL-6 production in M(IL-4/IL-13) polarized macrophages by differential miR-143/miR-365 targeting. **(A)** BMDMs were transfected with antisense oligonucleotides (GapmerRs) against c-Maf, Bach-1, and Elmo-1 alone or **(B)** in combination with MiR-143 or MiR-365 mimics followed by IL-4/IL-13 stimulation and infection with Mtb HN878. Macrophages were lysed at 4 h for uptake and 24 h post-Mtb infection to measure intracellular bacterial growth by CFU assay. **(C,D)** BMDMs were treated as described in **(B)** and culture supernatants were collected at 48 h post-infection to measure CCL5 chemokine, IL-6 and TNFα cytokines by ELISA. Data represented are mean ± SD of triplicates. A two-way ANOVA and Bonferroni *post-hoc* test was used to evaluate statistical significance. *P*-values represented as, ^*^P < 0.05, ^**^P < 0.01, ^***^P < 0.001, and ^****^P < 0.0001.

To examine the involvement of c-Maf, Bach-1, and Elmo-1 in Mtb-growth promotion within M(IL-4/IL-13) BMDMs, we knocked-down these target genes using GapmeRs in M(IL-4/IL-13) BMDMs prior to infection with Mtb (Figure 7A). Blocking of c-Maf resulted in increased intracellular Mtb growth when compared to control treated in M(IL-4/IL-13) BMDMs. Meanwhile, the blocking of Bach-1 had no effect and blocking Elmo-1 resulted in decreased Mtb growth. To further decrease the expression of these target genes and to demonstrate a direct link with miR-143 or miR-365 we treated macrophages with both mimics for miR-143 or miR-365 and the GapmeRs to c-Maf, Bach-1 and Elmo-1 target genes (Figure 7B). We found that Mtb CFU counts were restored when treated with both the GapmeRs for the target genes and mimics for miR-143. Conversely, CFU were increased when treated with GapmeRs and mimics for miR-365, thus suggesting the Mtb growth-promoting activities of miR-143 and miR-365 are mediated at least partially through interaction with c-Maf, Bach-1, and Elmo-1.

We then investigated the combined effect of miR-143/miR-365 mimics and anti-c-Maf/anti-Bach-1/anti-Elmo-1 treatment on CCL5, IL-6, and TNFα production. We observed that combined treatment of M(IL-4/IL-13) BMDMs with miR-143 or miR-365 mimics and anti-Bach-1/anti-Elmo-1 GapmeRs resulted in a significant decrease in IL-6 and CCL-5 production whereas treatment with anti-c-Maf and miR-143 or miR-365 mimics enhanced the expression of CCL-5 and IL-6, respectively. In contrast, TNFα levels were not affected (Figures 7C,D).

To further investigate the link between miR-143, miR-365, c-Maf, Bach-1, Elmo1, and IL-6/CCL5/TNF production, we used the online tool LASAGNA to search for the presence of binding sites for the transcription factors c-Maf and Bach-1 (TFBS) on the promoter regions of several genes of interest to our study, including IL-6, CCL5, TNFα, and IL-10. We found TFBS for Bach-1 and c-Maf on the promoter of IL-6 (Scores: 9.88; 6.46 and 5.15 for Bach-1 and 8.45 and 7.49 for c-Maf) and CCL-5 (Scores: 9.34 and 8.32 for Bach-1 and 9.16 and 6.49 for c-Maf). We found TFBS for c-MAF on the promoter of IL-10 (Scores: 9,32 for c-Maf), consistent with the known regulation of IL-10 by c-Maf. We found no TFBS for Bach-1 on the promoter of TNFα and a score below 8 for c-Maf (Table S1). This *in-silico* result indicates that IL-6 and CCL-5 production are most likely regulated by Bach-1 and our wet lab experiments suggest IL-6 and CCL-5 are indirectly regulated by miR-143 and miR-365 at least through Bach-1 and c-Maf.

## Discussion

Macrophage polarization and activation are critical for their anti-microbial activity. Classical activation of macrophages by IFN-γ/LPS results in concomitant production of reactive nitrogen species (RNIs), such as nitric oxide (NO), a free radical that kills intracellular Mtb (32). In contrast, alternative activation of macrophages is induced by IL-4 and/or IL-13, with increased arginase 1 expression and decreased levels of NO. Virulent and clinical Mtb strains can further induce Arg1 expression, potentially thriving in alternatively activated macrophages to establish persistence and survival (33). Our data highlight the host detrimental role of miR-143 and miR-365 that we found to be highly induced in Mtb (HN878)-infected M(IL-4/IL-13) polarized mouse and human macrophages. Previous reports have observed a significant increase of miR-365 in peripheral blood mononuclear cells (29) and of miR-365 and miR-143 in serum (8) from patients with active pulmonary tuberculosis as compared to matched healthy controls. Conversely, miR-365 expression was significantly lower in alveolar macrophages from patients with active pulmonary TB as compared to healthy controls, which inversely correlated with IL-6 heightened expression (34). MiR-143-3p, was also found to be 2-fold up-regulated in CD4^+^ T-cells from latent vs. active TB patients (35). These correlative studies suggest miR-143 and miR-365 may be important in the context of Mtb infection, prompting for further functional validation analysis. Depletion of miR-143 and miR-365 using antagomiRs reduced the mycobacterial intracellular growth and promoted the apoptotic death of Mtb-infected M(IL-4/IL-13) macrophages, hence supporting a possible role of miR-143 and miR-365 in promoting the intracellular Mtb growth via inhibition of the apoptotic death of Mtb-infected M(IL-4/IL-13) macrophages.

MiR-365 was previously identified as a direct negative regulator of IL-6 in HeLa and HEK293 cells (20), whereas the gene cluster miR-143/145 was reported to target KLF4 in smooth muscle cells (31). KLF4 is a key transcription factor that regulates macrophage polarization and the expression of CCL5 and TNFα (30). Given the crucial role of IL-6, CCL5, and TNFα in the control of Mtb infection, we sought to examine whether knocking down miR-143 and miR-365 would result in altered production of the above mentioned subset of pro-inflammatory cytokines/chemokine. Anti-miR-143 treatment markedly decreased CCL5 release by Mtb-infected M(IL-4/IL-13) BMDMs and had no effect on the production of TNFα. Our results suggest the effect of miR-143 on KLF4 may not be involve in the regulation of CCL5 and TNFα in M(IL-4/IL-13) BMDMs as compared to smooth muscle cells (31). In addition, our CAGE data disqualify KLF4 as a target of miR-143 in M(IL-4/IL-13) BMDMs as there is no inverse correlation between miR-143 and KLF4 expressions in M(IL-4/IL-13) BMDMs (Figure S2A). Moreover, KLF4 was not identified as one of the top miR-143 target genes by neither of the target prediction algorithms used (mirRanda, Target Scan, picTar). It is well-known that targets and functions of miRNAs may differ drastically in different cell types (36). Further, anti-miR-365 treatment also exhibited no effect on the expression of TNFα but significantly decreased CCL5 production by Mtb-infected M(IL-4/IL-13) BMDMs. Knockdown of miR-365 significantly decreased IL-6 levels in Mtb-infected M(IL-4/IL-13) BMDMs, suggesting that miR-365 does not act as a negative regulator of IL-6 in M(IL-4/IL-13) BMDMs during Mtb infection. This result is contrary to the findings by Xu et al. which showed that inhibition of miR-365 slightly increased IL-6 levels in HEK93 cells (20). Again, this highlights the cell type specificity of miRNA action. As diminished IL-6 levels are also exhibited with knockdown of miR-143 it can be concluded that this reduction is not specific to either miRNA. Hence, miR-143 and miR-365 enhances production of IL-6 and CCL5 through indirect mechanisms in M(IL-4/IL-13) macrophages.

We therefore sought to identify direct target genes of miR-143 and miR-365 which may expand the spectrum of their mechanisms of action in M(IL-4/IL-13) macrophages. It is worth mentioning that both miR-143 and miR-365 play a role in cancer onset and development by targeting distinct biological processes that are also involved in immune response to diseases (37–45). We identified *in silico* 2 genes (Ahcyl-1; Top2a) potentially targeted by miR-143; 1 gene potentially targeted by miR-365 (Igf-1) and 3 genes (Elmo-1; c-Maf; Bach-1) potentially targeted by both miR-143 and miR-365. There is a body of evidence supporting the notion of miRNA circuity whereby a single miRNA will regulate many genes involved in the same biological process and conversely, different miRNAs may regulate the same biological process by targeting the same mRNA. For functional validation experiments we focused on c-Maf, Bach-1 and Elmo-1 because they were predicted by at least two miRNA-target predictions algorithms as possible targets for both miR-143 and miR-365. Luciferase reporter assay, RNA pulldown assays and western blotting established that c-Maf, Bach-1 and Elmo-1 are directly targeted by miR-365 meanwhile, c-Maf and Bach-1 are directly targeted by miR-143 in macrophages.

c-Maf (V-maf musculoaponeurotic fibrosarcoma oncogene homolog) is a transcription factor that has been reported to suppress expression of pro-inflammatory cytokines including IL-12p35 (46) meanwhile promoting the expression of anti-inflammatory IL-10 (47) and hyaluronan synthase 1 (Has-1), two factors that promote growth of H37Rv Mtb strain in the CD14^high^ subset of human MDMs (48). Our CAGE data showed a lower expression of Has-1 in Mtb-infected M(IL-4/IL-13) BMDMs when compared to Mtb-infected M0 BMDMs (Figure S2B), which might result from c-Maf downregulation. Bach-1 (BTB and CNC homology 1) is a transcription factor whose expression was attenuated by miR-155 in Mtb-infected mouse macrophages (49). Bach-1 was also shown to repress the expression of antioxidant enzyme heme oxygenase-1 (HO-1) (50) which promotes Mtb survival in macrophage (51). Our CAGE data show a higher expression of HO-1 in Mtb-infected M(IL-4/IL-13) BMDMs when compared to Mtb-infected M0 BMDMs (Figure S2C). This heightened HO-1 expression might partially result from miR-143/miR-365-mediated downregulation of Bach-1 and could explain the drop in Mtb growth observed in M(IL-4/IL-13) BMDMs treated with miR-143/miR-365 antagomirs. Elmo-1 (Engulfment and cell motility protein 1) was reported to promote phagocytosis via cytoskeletal rearrangements. It also acts in association with DOCK1 and CRK to improve the efficiency of phagocytosis of dead cells by the phagocytic fibroblast line LR73 (52). Recently, ELMO1 was reported to favor the internalization and sensing of *Salmonella typhimurium* into enteric macrophages as well as induction of inflammatory molecules, such as MCP-1, TNF-α, keratinocyte-derived chemokine (KC), and RANTES (53). Moreover, Elmo-1 was shown to enhance bacterial clearance by regulating LC3-associated phagocytosis of *Salmonella typhimurium* in enteric macrophages (54). The role of Elmo-1 in Mtb infection has not been investigated so far.

Knockdown of c-Maf promoted intracellular Mtb growth when compared to control treated M(IL-4/IL-13) macrophages. Meanwhile, the blocking of Bach-1 had no effect and blocking Elmo-1 resulted in decreased Mtb growth. Combination treatment of M(IL-4/IL-13) macrophages with miR-143 mimics or miR-365 mimics and c-Maf, Bach-1 and Elmo-1 gene-specific GapmeRs restored Mtb growth in miR-143 mimics-treated groups and enhanced Mtb growth in miR-365 mimics-treated groups, thus suggesting the Mtb growth-promoting activities of miR-143 and miR-365 are mediated at least partially through interaction with c-Maf, Bach-1 and Elmo-1. Our *in-silico* and *in-vitro* experiments further demonstrated that miR-143 and miR-365 could promote Mtb growth and survival through Bach-1 and c-Maf-mediated enhancement of IL-6 and CCL-5 production in M(IL-4/IL-13) macrophages. We further show that knockdown of miR-143 and miR-365 in M(IL-4/IL-13) BMDM decreased the expression of HO-1 (Figure S3A) and IL-10 (Figure S3B) which are known targets of Bach-1 and c-Maf, respectively, with Mtb growth-promoting activities in macrophages.

In summary, we have identified miR-143 and miR-365 as highly up-regulated in Mtb-infected M(IL-4/IL-13) polarized macrophages. Knockdown of miR-143 and miR-365 decreased mycobacterial burden and significantly reduced the release of chemokine CCL5 and pro-inflammatory cytokine IL-6 in M(IL-4/IL-13) macrophages. We validated c-Maf, Bach-1, and Elmo-1 as potential target genes for both miRNAs. Altogether, our work reports a host detrimental role of miR-143 and miR-365 during Mtb infection and highlights for the first time the role of miRNA-mediated regulation of c-Maf, Bach-1 and Elmo-1 in Mtb-infected M(IL-4/IL-13) macrophages. These results expand the possible roles for miR-143, miR-365, c-Maf, Bach-1, and Elmo-1 in the control of Mtb growth in alternative M(IL-4/IL-13) activated macrophages and urges for further investigation of these miRNAs and target genes as potential candidate for host directed therapy against TB.

## Ethics Statement

The BALB/c mouse strain were bred and housed in specific pathogen-free conditions and all animal procedures were performed in compliance with the standards of practice for laboratory animal procedures set by the Animal Research Ethics Committee, Faculty of Health Sciences, University of Cape Town, Cape Town, South Africa (Ethics approval Ref 015/037). The recruitment of healthy volunteers for this study was approved by the Human Ethics Committee, Faculty of Health Sciences, University of Cape Town, Cape Town (HREC Ref Number: 635/2015). Inclusion criteria were as follows: age 18–50 years, both sexes, no history of TB, no contact with TB patients, HIV negative, sputum smear-negative, non-smokers, no chronic alcoholism, normal chest X-rays, no chronic disease, not receiving immunosuppressive therapy, IGRA negative and absence of other pulmonary diseases. The participants who did not meet the above criteria did not consent to signing the informed consent form or to undertaking a HIV test were excluded from this study.

## Author Contributions

OT, RG, and FB conceived and designed the experiments. OT, LG, LW, MO, SP, RJ, and MD performed the experiments. OT, LG, RG, SR, SS, MD, YM, KD, and HS analyzed the data. OT, LG, LW, and RG wrote the paper. All of the authors read and approved the final manuscript.

### Conflict of Interest Statement

The authors declare that the research was conducted in the absence of any commercial or financial relationships that could be construed as a potential conflict of interest.

## Supplementary Material

Figure S1 MiR-143 and miR-365 are upregulated in Mtb HN878-infected M(IL-4/IL-13) activated human macrophages and their knockdown decreases intracellular Mtb HN878 intracellular growth. **(A)** MDMs were stimulated with IL-4/IL-13. At 24 h post-stimulation, MDMs were infected with Mtb HN878 for 4 and 24 h. RNA was extracted from lysed cells at different time points post-Mtb infection and the fold change in gene expression was determined by RT-qPCR and normalized to miR-191 expression. Non-stimulated 0 h was set to 1. Each data point represents arithmetic mean of triplicates ± SEM. **(B)** MDMs were transfected with antagomiRs for miR-143 and miR-365, respectively. Twenty-four hours later, cells were stimulated with IL-4/IL-13 for another 24 h and subsequently infected with Mtb HN878. Cells were lysed at 4 h for uptake and 24 h post-Mtb infection to measure bacterial growth by CFU counting. Data represented here are mean ± SD of triplicates. A two-way ANOVA and Bonferroni *post-hoc* test was used to evaluate statistical significance. *P*-values represented as, ^*^*P* < 0.05, ^**^*P* < 0.01, and ^***^*P* < 0.001.

Figure S2 CAGE expression data for KLF-4, HAS-1, and HO-1 genes. Gene expression level (Tags Per Million) for **(A)** Kruppel-like Factor 4 (known target of miR-143), **(B)** Hyaluronan synthase-1 (known target of c-Maf), and **(C)** Haem oxygenase-1 (known target of Bach-1) were plotted. These data were previously generated in collaboration with the Fantom5 consortium (24) where BMDMs were classically (IFNγ) or alternatively (IL-4 and IL-13) activated then infected with the hypervirulent Mtb HN878 strain for different duration. RNA samples were collected and analyzed by CAGE transcriptomics.

Figure S3 MiR-143 and miR-365 control the expression of HO-1, IL-10 genes in M(IL-4/IL-13) Mtb-infected mouse macrophages and miR-143/miR-365 expression is Mtb virulent dependent. **(A,B)** BMDMs were transfected with scramble, anti-miR-143 or anti-miR-365 antagomiRs. At 24 h post-transfection, macrophages were stimulated with a combination of IL-4/IL-13 for another 24 h then infected with Mtb HN878 for an additional 24 h. Fold change in gene expression of HO-1 and IL-10 was determined by RT-qPCR and normalized to Hprt1 expression. Non-stimulated 0 h was set to 1. Data represent the mean ± SD of triplicate. Two-way ANOVA was used to evaluate statistical significance, *P*-values represented as, ^*^*P* < 0.05, ^**^*P* < 0.01, and ^***^*P* < 0.001. **(C)** BMDMs were stimulated with a combination of IL-4/IL-13 for 24 h then infected with Mtb HN878 or Mtb H37Rv MOI of 5 bacilli: 1 cell (5:1) for another 24 h. Fold change in gene expression of HO-1 and IL-10 was determined by RT-qPCR and normalized to Hprt1 expression. Non-stimulated 0 h was set to 1. Data represent the mean ± SD of triplicate. Two-way ANOVA was used to evaluate statistical significance, *P*-values represented as, ^*^*P* < 0.05 and ^**^*P* < 0.01.

Table S1 Bach-1 and c-Maf binding sites on the promoters of target genes. The analysis of transcription factors (c-Maf and Bach-1) binding sites on the promoters of our target genes was done using the online free tool LASAGNA (http://biogrid-lasagna.engr.uconn.edu/lasagna_search/) using the following parameters: Search restricted to mus musculus; cut off p-value set to 0.005; score cut off set at 5; reporting of the top 5 TFBS.
