# Supplementary material for: Differential Targeting of c-Maf, Bach-1, and Elmo-1 by microRNA-143 and microRNA-365 Promotes the Intracellular Growth of Mycobacterium tuberculosis in Alternatively IL-4/IL-13 Activated Macrophages
Source: Front Immunol. 2019 Mar 19;10:421. doi: 10.3389/fimmu.2019.00421 (PMC6433885; doi:10.3389/fimmu.2019.00421)
Supplement: Figure S1 — MiR-143 and miR-365 are upregulated in Mtb HN878-infected M(IL-4/IL-13) activated human macrophages and their knockdown decreases intracellular Mtb HN878 intracellular growth. (A) MDMs were stimulated with IL-4/IL-13. At 24 h post-stimulation, MDMs were infected with Mtb HN878 for 4 and 24 h. RNA was extracted from lysed cells at different time points post-Mtb infection and the fold change in gene expression was determined by RT-qPCR and normalized to miR-191 expression. Non-stimulated 0 h was set to 1. Each data point represents arithmetic mean of triplicates ± SEM. (B) MDMs were transfected with antagomiRs for miR-143 and miR-365, respectively. Twenty-four hours later, cells were stimulated with IL-4/IL-13 for another 24 h and subsequently infected with Mtb HN878. Cells were lysed at 4 h for uptake and 24 h post-Mtb infection to measure bacterial growth by CFU counting. Data represented here are mean ± SD of triplicates. A two-way ANOVA and Bonferroni post-hoc test was used to evaluate statistical significance. P-values represented as, *P < 0.05, **P < 0.01, and ***P < 0.001. [file Presentation_1.PPTX]

## Slide 1
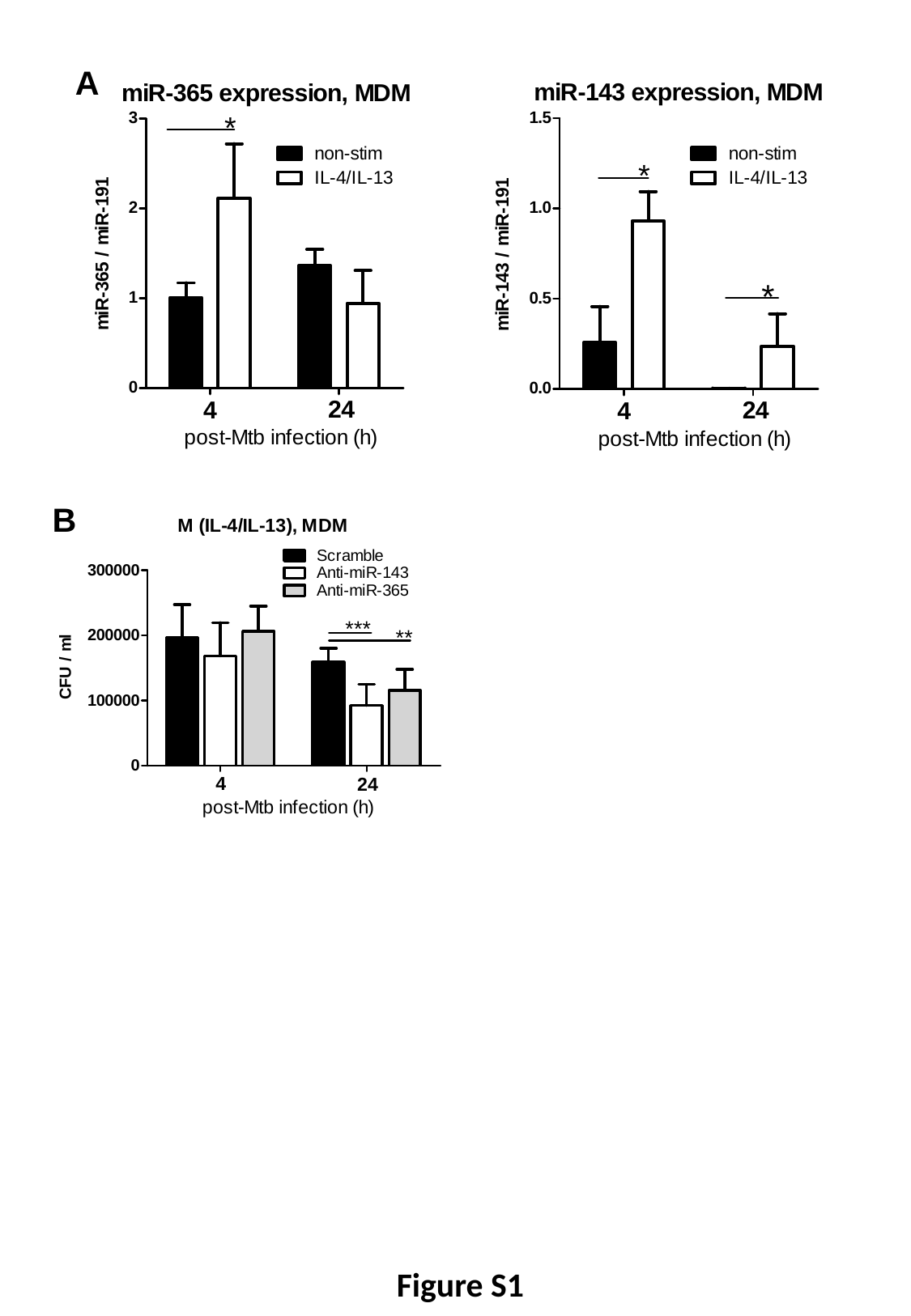

Figure S1

## Slide 2
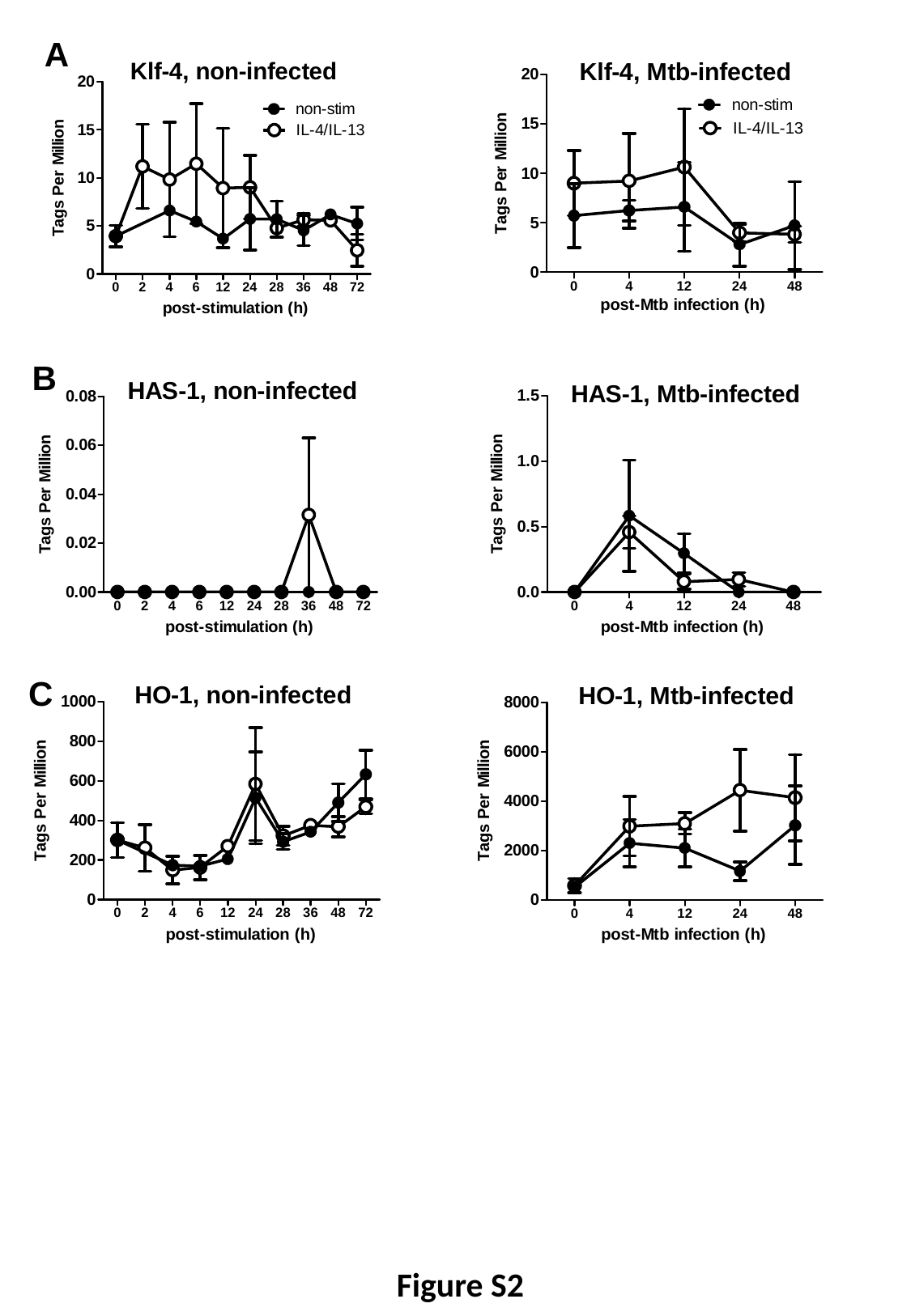

Figure S2

## Slide 3
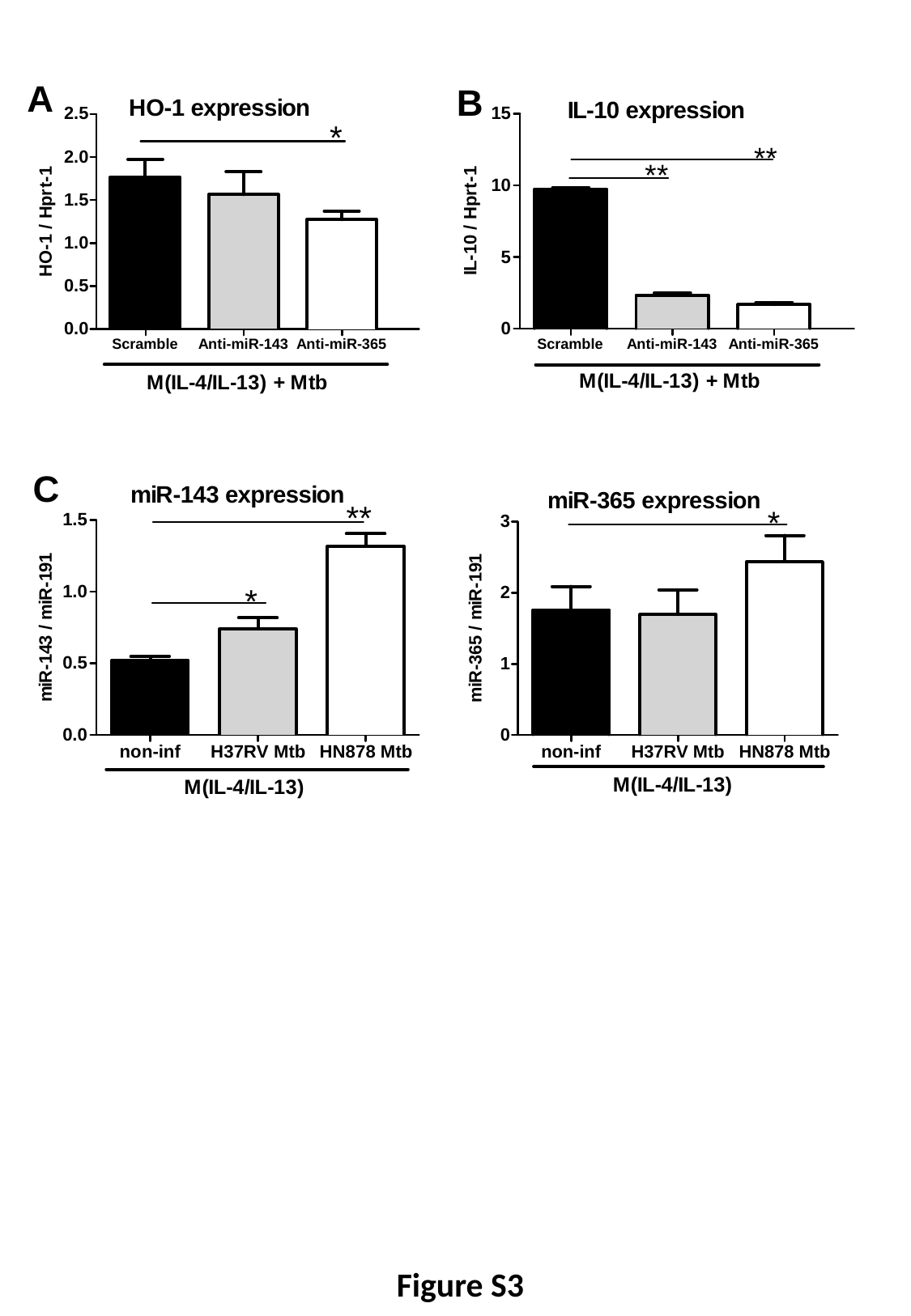

Figure S3

## Slide 4
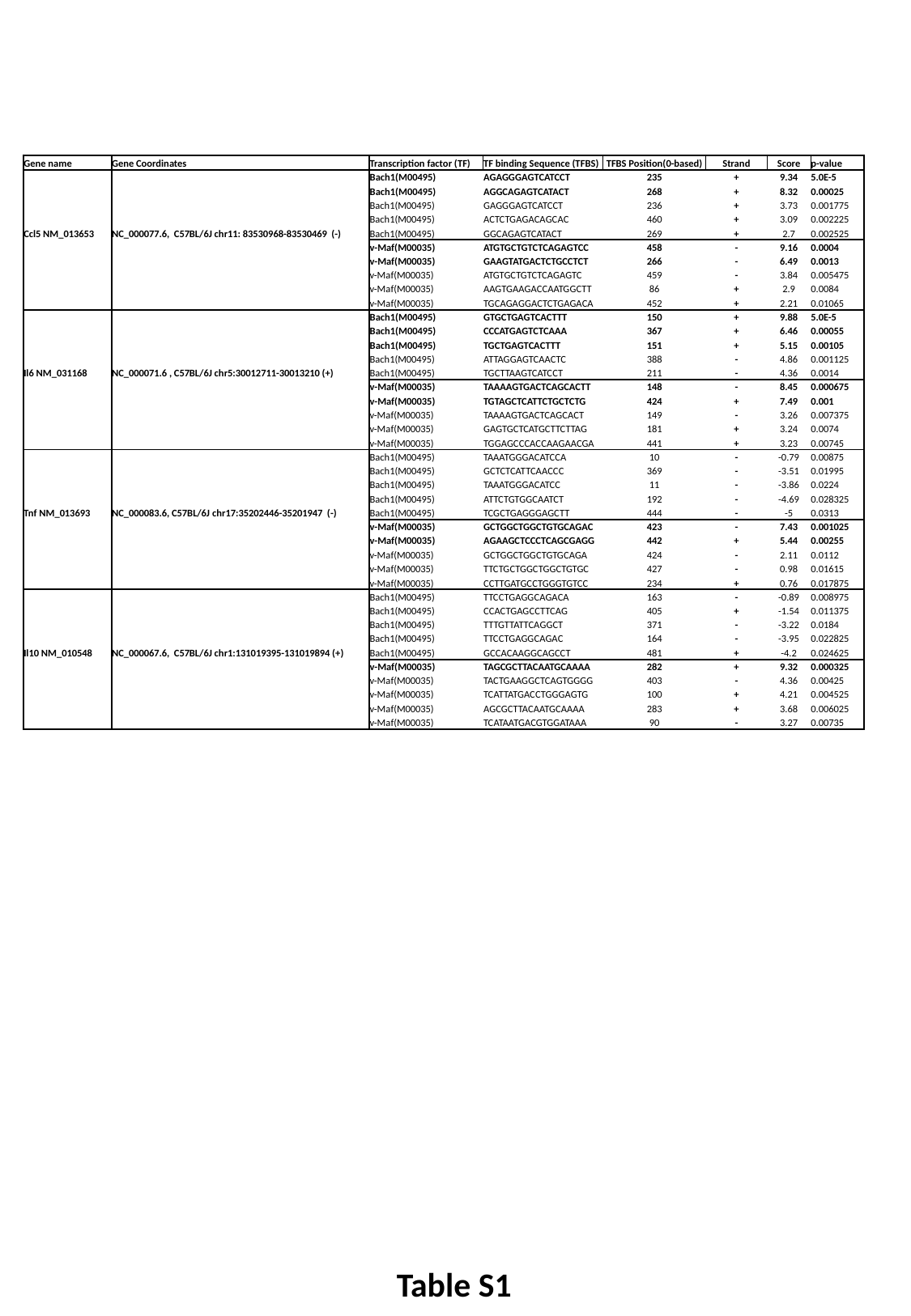

| Gene name | Gene Coordinates | Transcription factor (TF) | TF binding Sequence (TFBS) | TFBS Position(0-based) | Strand | Score | p-value |
| --- | --- | --- | --- | --- | --- | --- | --- |
| | | Bach1(M00495) | AGAGGGAGTCATCCT | 235 | + | 9.34 | 5.0E-5 |
| | | Bach1(M00495) | AGGCAGAGTCATACT | 268 | + | 8.32 | 0.00025 |
| | | Bach1(M00495) | GAGGGAGTCATCCT | 236 | + | 3.73 | 0.001775 |
| | | Bach1(M00495) | ACTCTGAGACAGCAC | 460 | + | 3.09 | 0.002225 |
| Ccl5 NM\_013653 | NC\_000077.6, C57BL/6J chr11: 83530968-83530469 (-) | Bach1(M00495) | GGCAGAGTCATACT | 269 | + | 2.7 | 0.002525 |
| | | v-Maf(M00035) | ATGTGCTGTCTCAGAGTCC | 458 | - | 9.16 | 0.0004 |
| | | v-Maf(M00035) | GAAGTATGACTCTGCCTCT | 266 | - | 6.49 | 0.0013 |
| | | v-Maf(M00035) | ATGTGCTGTCTCAGAGTC | 459 | - | 3.84 | 0.005475 |
| | | v-Maf(M00035) | AAGTGAAGACCAATGGCTT | 86 | + | 2.9 | 0.0084 |
| | | v-Maf(M00035) | TGCAGAGGACTCTGAGACA | 452 | + | 2.21 | 0.01065 |
| | | Bach1(M00495) | GTGCTGAGTCACTTT | 150 | + | 9.88 | 5.0E-5 |
| | | Bach1(M00495) | CCCATGAGTCTCAAA | 367 | + | 6.46 | 0.00055 |
| | | Bach1(M00495) | TGCTGAGTCACTTT | 151 | + | 5.15 | 0.00105 |
| | | Bach1(M00495) | ATTAGGAGTCAACTC | 388 | - | 4.86 | 0.001125 |
| Il6 NM\_031168 | NC\_000071.6 , C57BL/6J chr5:30012711-30013210 (+) | Bach1(M00495) | TGCTTAAGTCATCCT | 211 | - | 4.36 | 0.0014 |
| | | v-Maf(M00035) | TAAAAGTGACTCAGCACTT | 148 | - | 8.45 | 0.000675 |
| | | v-Maf(M00035) | TGTAGCTCATTCTGCTCTG | 424 | + | 7.49 | 0.001 |
| | | v-Maf(M00035) | TAAAAGTGACTCAGCACT | 149 | - | 3.26 | 0.007375 |
| | | v-Maf(M00035) | GAGTGCTCATGCTTCTTAG | 181 | + | 3.24 | 0.0074 |
| | | v-Maf(M00035) | TGGAGCCCACCAAGAACGA | 441 | + | 3.23 | 0.00745 |
| | | Bach1(M00495) | TAAATGGGACATCCA | 10 | - | -0.79 | 0.00875 |
| | | Bach1(M00495) | GCTCTCATTCAACCC | 369 | - | -3.51 | 0.01995 |
| | | Bach1(M00495) | TAAATGGGACATCC | 11 | - | -3.86 | 0.0224 |
| | | Bach1(M00495) | ATTCTGTGGCAATCT | 192 | - | -4.69 | 0.028325 |
| Tnf NM\_013693 | NC\_000083.6, C57BL/6J chr17:35202446-35201947 (-) | Bach1(M00495) | TCGCTGAGGGAGCTT | 444 | - | -5 | 0.0313 |
| | | v-Maf(M00035) | GCTGGCTGGCTGTGCAGAC | 423 | - | 7.43 | 0.001025 |
| | | v-Maf(M00035) | AGAAGCTCCCTCAGCGAGG | 442 | + | 5.44 | 0.00255 |
| | | v-Maf(M00035) | GCTGGCTGGCTGTGCAGA | 424 | - | 2.11 | 0.0112 |
| | | v-Maf(M00035) | TTCTGCTGGCTGGCTGTGC | 427 | - | 0.98 | 0.01615 |
| | | v-Maf(M00035) | CCTTGATGCCTGGGTGTCC | 234 | + | 0.76 | 0.017875 |
| | | Bach1(M00495) | TTCCTGAGGCAGACA | 163 | - | -0.89 | 0.008975 |
| | | Bach1(M00495) | CCACTGAGCCTTCAG | 405 | + | -1.54 | 0.011375 |
| | | Bach1(M00495) | TTTGTTATTCAGGCT | 371 | - | -3.22 | 0.0184 |
| | | Bach1(M00495) | TTCCTGAGGCAGAC | 164 | - | -3.95 | 0.022825 |
| Il10 NM\_010548 | NC\_000067.6, C57BL/6J chr1:131019395-131019894 (+) | Bach1(M00495) | GCCACAAGGCAGCCT | 481 | + | -4.2 | 0.024625 |
| | | v-Maf(M00035) | TAGCGCTTACAATGCAAAA | 282 | + | 9.32 | 0.000325 |
| | | v-Maf(M00035) | TACTGAAGGCTCAGTGGGG | 403 | - | 4.36 | 0.00425 |
| | | v-Maf(M00035) | TCATTATGACCTGGGAGTG | 100 | + | 4.21 | 0.004525 |
| | | v-Maf(M00035) | AGCGCTTACAATGCAAAA | 283 | + | 3.68 | 0.006025 |
| | | v-Maf(M00035) | TCATAATGACGTGGATAAA | 90 | - | 3.27 | 0.00735 |
Table S1
